# Prevalence and predictors of anxiety and depressive symptoms among patients diagnosed with oral cancer in China: a cross-sectional study

**DOI:** 10.1186/s12888-020-02796-6

**Published:** 2020-08-05

**Authors:** Lulu Yuan, Bochen Pan, Weiren Wang, Lie Wang, Xujie Zhang, Yuqin Gao

**Affiliations:** 1 Department of Nursing, School and Hospital of Stomatology, China Medical University, Liaoning Provincial Key Laboratory of Oral Diseases, Shenyang, P.R. China; 2 Shengjing Hospital, China Medical University, Shenyang, P.R. China; 3 School of Public Health, China Medical University, Shenyang, P.R. China

**Keywords:** Oral cancer, Anxiety symptoms, Depressive symptoms

## Abstract

**Background:**

Anxiety and depression are common mental health problems among patients with cancer. While many psychological variables have been proven to influence anxiety and depressive symptoms, the variables are not mutually exclusive and their integrated effects on patients with oral cancer are yet unknown. The present study aims to explore the prevalence of anxiety and depressive symptoms among patients with oral cancer, to find out key potentially predictive factors associated with anxiety and depressive symptoms.

**Method:**

A cross-sectional study was carried out for Chinese patients with oral cancer between May 2016 and October 2017 in two Grade-A Tertiary Hospitals in Shenyang, China. Two hundred thirty patients with oral cancer were interviewed with questionnaires on demographic variables, Zung Self-Rating Anxiety Scale (SAS), Center for Epidemiologic Studies Depression Scale (CES-D), Herth Hope Index (HHI), Social Impact Scale, Multidimensional Scale of Perceived Social Support (MSPSS), Revised Life Orientation Test (LOT-R), Perceived Stress Scale-10 (PSS-10), and General Perceived Self-efficacy Scale(GSE). Chi-square test, nonparametric test, t-test and logistic regression analyses were conducted where appropriate to explore predictive factors of anxiety symptoms and depressive symptoms.

**Results:**

The prevalence of anxiety symptoms and depressive symptoms in the sample population was 36.96% (85/230) and 65.21% (150/230), respectively. Social isolation dimension of stigma (β = 0.436, OR = 1.547, CI:1.211 ~ 1.975), optimism (β = − 0.276, OR = 0.759, CI:0.624 ~ 0.922), and perceived stress (β = 0.217, OR = 1.243, CI:1.092 ~ 1.414) were predictors of anxiety symptoms. Marriage (β = 1.648, OR = 5.198, CI:1.427 ~ 18.924), positive readiness and expectancy dimension of hope (β = − 0.505, OR = 0.604, CI:0.395 ~ 0.923), social isolation dimension of stigma (β = 0.314, OR = 1.368, CI:1.054 ~ 1.776) and perceived stress (β = 0.273, OR = 1.314, CI:1.134 ~ 1.524) were predictors of depressive symptoms among oral cancer patients.

**Conclusion:**

The prevalence of anxiety symptoms and depressive symptoms was high among oral cancer patients in China. The communal predictors of anxiety and depressive symptoms in patients with oral cancer were levels of perceived stress and social isolation of stigma. In addition, optimism was a predictor of anxiety symptoms and hope was a predictor of depressive symptoms.

**Trial registration:**

2015–16, registered 20 Dec 2015.

## Background

Oral cancer is a broad term of the oral cavity and oropharyngeal cancers such as floor of mouth, palate, cheek, lip and parotid gland carcinomas. Global data shows that over 350,000 cases of oral cavity cancer are diagnosed worldwide and roughly about 180,000 die from it every year [1]. It is universally acknowledged that the diagnosis of cancer is a huge stress for both individuals and families, which can exert substantial effects on the development of anxiety and depression [2]. Anxiety and depression can interfere with the ability to adapt to the stress of life-threatening illnesses. For instance, the length of hospitalization, treatment compliance, quality of life and survival time are all compromised as a result of such problems for patients with cancer [3–5]. Previous studies have shown that there is a high prevalence of anxiety and depressive symptoms among different types of patients with cancer in China [6–8]. However, Hong and Tian reported that the prevalence of depressive symptoms among patients with head and neck cancer was as high as 60.62%, while that of anxiety was 1.33% in mainland China, which was rather confusing [9].

Several factors that have been reported related to the occurrence of anxiety and depression among patients with cancer. Studies have shown that factors such as age, gender, education level and others have significant associations with the negative moods among patients [10–13]. In addition, stigma, “an attribute that is deeply discrediting”, is regarded as a mark that reduces the sufferer “from a whole and usual person to a trained, discounted one” [14]. Stigma in cancer patients has been found to be strongly and consistently associated with poor mental health, including depressive symptoms [15], anxiety [16], and demoralization [17]. Furthermore, studies conducted in the field of health psychology have started to explore the effects of positive psychological resources such as hope, self-efficacy, optimism, and social support in order to explain differences in anxiety and depressive symptoms among cancer patients. Hope is “a multidimensional dynamic life force characterized by a confident yet uncertain expectation of achieving a good future which, to the hoping person, is realistically possible and personally significant” [18]. General self-efficacy (GSE) [19] is the individual’s subjective perception in his capacity to deal with various stressful situations, like coping with cancer, its treatments, and numerous side- or late- effects. Individuals with high GSE believe in themselves with the competence to mobilize the behavioral, cognitive and motivational resources required to cope with the situation [19]. Optimism is a personality trait characterized by a general tendency to hold positive expectations about the future that functions as a psychological resource conferring health benefit [20]. Social support is defined as the material and moral support provided to the individual under stress or in a difficult condition by the people around him/her [21]. The aforementioned psychological resources have been shown to have positive effects on anxiety and depression in patients with most chronic illnesses, including cancer [22–25].

As researchers have increasingly recognized the value of mental health of individuals with cancer, alleviating symptoms of anxiety and depression has been an important challenge, and exploring the relevant psychosocial factors of anxiety and depressive symptoms so as to provide essential psychological support is of vital necessity. While these negative and positive psychological variables mentioned above have effects on emotional issues of cancer individuals, they are not mutually exclusive and their integrated effects on oral cancer patients are yet unknown, especially in patients with oral cancer. We propose the hypotheses that anxiety and depressive symptoms are negatively associated with perceived stress and stigma and positively associated with perceived social support, self-efficacy, optimism, and hope. The aim of the current study is to explore the prevalence of anxiety and depressive symptoms in oral cancer patients and to find out key factors that have potential predictive value for anxiety and depressive symptoms.

## Methods

### Settings of the study

This cross-sectional study was conducted in two Grade-A Tertiary Hospitals in Shenyang, located in northeast China. Both are provincial public hospitals affiliated to medical universities. The first is a stomatological hospital, and the second is a general hospital. Data were collected from inpatients in oral and maxillofacial surgery ward between May 2016 and October 2017. The current research was approved by the Ethical Committee of China Medical University (NO. 2015–16).

### Subjects

The inclusion criteria were: patients (1) aged 18 or above; (2) had been diagnosed with oral cancer for the first time; (3) had finished the surgeries; (4) were aware of their own diagnosis; (5) the condition was good enough to understand and complete the questionnaires. The exclusion criteria were that patients (1) with any history of mental or cognitive disorders; (2) were comorbid with other oral diseases or other cancers. Each participant was limited to completing the survey only once. The study size was arrived at by using the following formula: $n=\frac{z_{\alpha}^2{\sigma}^2}{\delta^2}$. The parameters were: α = 0.05, Zα = 1.96, σ = 14.52, δ = 2. *n* = 1.96^2^*14.52^2^/2^2^ = 202.48. Considering that there were invalid questionnaires or lost follow-up, the sample size was increased by 10% ~ 20%, and the final sample size was 224 ~ 243.6.

### Procedure

The whole process of the study was anonymous and voluntary for respondents. Investigators consisted of four nurses, whom were trained uniformly by the researcher. Before filling in the questionnaire, participants signed the consent inform. The investigators were responsible to read and provide explanations for questionnaire items without any inducement. Another trained investigator conducted quality control on the spot and then collected the questionnaires. Epidata software (version 3.1) was used for data entry and double check.

### Tools

Demographic and clinical characteristics composed of a general questionnaire. Demographic characteristics consisted of age, gender, body mass index (BMI), marital status, education level, monthly income, occupation, residence area, smoking, and alcohol consumption. Clinical variables were made up of patients’ type of treatment, family history and whether they had distant metastasis.

#### Measurement of anxiety symptoms

Zung Self-Rating Anxiety Scale (SAS) [26] was used to assess the anxiety symptoms of the patients. The SAS included 20 items, and each item was rated on a 4-point scale, with a total score ranging from 20 to 80, the standardized score = int (1.25*raw score). A higher score means more severe anxiety symptoms. SAS has been reported with good reliability and validity in China [27], and a standardized score of 50 was the upper limit for the normative populations [28]. The Cronbach’s α was 0.908 in the current study.

#### Measurement of depressive symptoms

Depressive symptoms were assessed with the Center for Epidemiologic Studies Depression Scale (CES-D) [29]. The CES-D is a 20-item tool rating on 4-point scoring system, with a total score ranging from 0 to 60. A total score of 16 or above was considered with depressive tendencies [30]. The Chinese version has been shown with good reliability and validity [30]. The Cronbach’s α was 0.924 in the current study.

#### Measurement of hope

Hope was assessed by the Herth Hope Index (HHI) [31], which contained 3 subscales: temporality and future, positive readiness and expectancy, and interconnectedness. The HHI consisted of 12 items, and each item was scored on a 4-point scale. Total score of HHI ranged from 12 to 48, and a higher total score reflected higher level of hope. The Chinese version of HHI had been found with good reliability and validity [32]. In the current study, the Cronbach’s α found to be 0.841.

#### Measurement of stigma

The Social Impact Scale (SIS) was developed to assess the level of stigmatization for individuals with cancer or HIV/AIDS [33]. The SIS is a 24-item scale, with 4 domains: social rejection, financial insecurity, internalized shame, and social isolation. Each item rated on 4-point scoring system, with a total score ranging from 24 to 96. The scale has been reported available in different populations [34]. In the current research, the Cronbach α of the SIS was 0.948.

#### Measurement of social support

The level of perceived social support was assessed by the Chinese version of the Multidimensional Scale of Perceived Social Support (MSPSS) [35] which measured perceived support from three social relationships: family, friends and significant others (such as relatives and colleagues). It included 12 items rated on 7-point scale. Total score ranged from 12 to 84, with a higher score indicating higher social support. The scale had good reliability and validity among various Chinese patients [36, 37]. In this study, the Cronbach’s α of the MSPSS was 0.928.

#### Measurement of optimism

Optimism was assessed by the a 10-item Revised Life Orientation Test (LOT-R), which was designed by Dr. Scheier et al. [38]. It consisted of ten items using 5-point rating system, three of which were for optimism; three of which were for pessimism; the other four items served as fillers. The Cronbach’s α was 0.646 in the current research.

#### Measurement of perceived stress

Perceived stress was assessed by the 10-item version of Perceived Stress Scale (PSS-10) [39]. Each item was scored using a 5-point scale, with a total score ranging from 0 to 40. Higher scores indicated higher level of perceived stress. The Chinese version has demonstrated good reliability and validity [40]. The Cronbach’s α was 0.833 in this study.

#### Measurement of self-efficacy

General Self-efficacy Scale (GSE) was used to assess the self-efficacy of the respondents [41]. The GSE was a 10-item scale rated on a 4-point scale, with a total score ranging from 10 to 40. Higher scores indicated a higher level of self-efficacy. The scale has been widely used among Chinese population [42]. The Cronbach’s α was 0.913 in the current study.

#### Operational definition

The cut-off points of SAS and CES-D were set to be the criteria to differentiate whether patients had symptoms of anxiety/depression. According to the previous studies [28, 30], patients with a 50 or above SAS standardized score were classified into the anxiety symptoms group, and patients with a CES-D score over 16 or above were defined as the depressive symptoms group.

### Statistical analyses

Statistical Package for Social Sciences (SPSS 22.0 for Windows) was used to conduct data analyses. Significance for all statistical tests was set to be the level of 0.05 (2-tailed). Normality and homogeneity of variances were first tested for each continuous variable. Chi-square test was operated to describe distributions of anxiety symptoms and depressive symptoms in categorical demographic and clinical variables. Independent sample T-test and nonparametric-test were used to explore the relationship between anxiety/depressive symptoms and the grouping variables (hope, social support, optimism, stigma, and perceived stress). Logistic regression analyses were conducted to find the predictors. The variables with P<0.2 in the Chi-square test and variables related to symptoms of anxiety and depression were entered into regression analysis in order to not overfit the logistic regression models [43]. And the independent variables (hope, perceived social support, optimism, stigma, and perceived stress) were also entered into the regression. Multicollinearity diagnostic tests were carried out by the variance inflation factor (VIF), Tolerance, Eigenvalue and Condition Index and Variance Proportions. Variables were entered in the regression analysis at *P* < 0.05 and removed from the model at *P* > 0.10. Data provided in the regression models included regression coefficient (β), OR, 95%CI.

## Results

### Descriptive statistics

In the current study, 275 questionnaires were distributed. Among them, 230 were considered valid, yielding an effective response rate of 83.64%. Altogether 134 male and 96 female patients participated.

All in all, 85 respondents reported anxiety symptoms, 150 reported depressive symptoms, and the prevalence was 36.96 and 65.21%, respectively. Furthermore, 84 patients reported both anxiety symptoms and depression symptoms.

The demographic and medical information of the participants were described in Table 1. The mean age of the respondents was 55.47 years (SD = 13.78, ranging from 18 to 92). Most patients (204, 88.7%) were in a married or cohabited status, In terms of the clinical variables, over 90% of the patients (215) reported a family history of cancer. Most patients were without metastasis (216, 94.0%).

**Table 1:** Distributions of anxiety symptoms and depressive symptoms in categorical demographic and clinical variables (*n* = 230)

	***N(%)***	***Anxiety symptoms***			***Depressive symptoms***		
		***No. (%)***	***×***^***2***^	***p***	***No. (%)***	***×***^***2***^	***p***
**Age**							
< 60	156(67.8)	57(36.5)	0.036	0.849	105(67.3)	0.934	0.334
≥ 60	74(32.2)	28(37.8)			45(60.8)		
**Gender**							
male	134(58.3)	49(36.6)	0.021	0.885	93(69.4)	2.479	0.115
female	96(41.7)	36(37.5)			57(59.4)		
**Marriage**							
Single/divorced /widow	26(11.3)	7(26.9)	1.267	0.260	10(38.5)	9.251	0.002
Married/cohabitation	204(88.7)	78(38.2)			140(68.6)		
**BMI**							
<18.5	8(3.5)	5(62.5)	2.803	0.246	6(75.0)	0.371	0.831
18.5–23.9	118(51.3)	40(33.9)			76(64.4)		
≥ 24	104(45.2)	40(38.5)			68(65.4)		
**Education**							
Middle school or lower	100(43.5)	33(33.0)	1.184	0.553	66(66.0)	0.253	0.881
High or secondary school	60(26.1)	24(40.0)			40(66.7)		
College or university	70(30.4)	28(40.0)			44(62.9)		
**Job state**							
Regular employee	133(57.8)	54(40.6)	2.039	0.361	89(66.9)	0.429	0.807
Retirement	34(14.8)	12(35.3)			21(61.8)		
Unemployed /temporary workers	63(27.4)	19(30.2)			40(63.5)		
**Income**							
< 3000	141(61.3)	56(39.7)	1.191	0.275	94(66.7)	0.337	0.561
≥ 3000	89(38.7)	29(32.6)			56(62.9)		
**Residence**							
Urban	145(63.0)	52(35.9)	0.267	0.605	92(63.4)	0.738	0.390
Rural	85(37.0)	33(38.8)			58(68.2)		
**Smoking**							
No	118(51.3)	43(36.4)	0.028	0.868	71(60.2)	2.722	0.099
Yes	112(48.7)	42(37.5)			79(70.5)		
**Drinking alcohol**							
No	135(58.7)	51(37.8)	0.095	0.752	86(63.7)	0.330	0.566
Yes	95(41.30)	34(35.8)			64(67.4)		
**Family history**							
No	215(93.5)	80(37.5)	0.090	0.764	138(64.2)	1.546	0.214
Yes	15(6.5)	5(33.3)			12(80.0)		
**Distant metastasis**							
No	216(94.0)	76(35.2)	4.779	0.029	138(63.9)	1.883	0.170
Yes	14(6.0)	9(64.3)			12(85.7)		

Analysis was performed with x^2^ test

*N* number, *BMI* Body Mass Index

### Distributions of anxiety and depressive symptoms in continuous variables

The distributions of anxiety symptoms and depressive symptoms in continuous variables including hope, stigma, self-efficacy, perceived social support, optimism, perceived stress were presented in Table 2. Results showed that the distribution of anxiety symptoms and depressive symptoms were significantly different in all the variables and its subscales (p<0.01). Both anxiety and depressive symptoms were negatively associated with hope and its subscales, perceived social support and its subscales, self-efficacy, optimism, but positively associated with stigma and its subscales, and the perceived stress(*p* < 0.01).

**Table 2:** Distributions of anxiety and depressive symptoms in continuous variables (*n* = 230, Median (IQR)/ (M ± SD))

	***Anxiety symptoms***				***Depressive symptoms***			
	Yes	***No***	***Z/t***	***p***	Yes	***No***	***Z/t***	***p***
	***N = 85***	***N = 145***			***N = 150***	***N = 80***		
**Hope**	35.00 (5.50)	37.00 (6.00)	−6.498	0.000	35.00 (5.00)	40(5.75)	−7.883	0.000
Temporality and future	11.00 (2.00)	12.00 (2.00)	−5.543	0.000	11.00 (2.00)	13.00 (2)	−7.144	0.000
Positive readiness and expectancy	12.00 (2.00)	12.00 (2.00)	−4.886	0.000	12.00 (2.00)	13.00 (2.75)	−5.835	0.000
Interconnectedness	12.00 (2.00)	13.00 (2.00)	−6.794	0.000	12.00 (2.00)	14.00 (2.00)	−7.557	0.000
**Social support**	58.00 (17.75)	65.00 (13.00)	−4.513	0.000	59.00 (17.00)	67.00 (10.75)	−4.847	0.000
Family support	21.00 (7.00)	24.00 (3.00)	−4.149	0.000	22.00 (6.00)	24.00 (2.00)	−3.579	0.000
Friend support	17.00 (6.00)	20.00 (7.00)	−3.511	0.000	17.00 (6.00)	20.00 (7.75)	−4.485	0.000
Other support	18.00 (7.00)	22.00 (5.00)	−4.646	0.000	19.00 (6.25)	23.00 (4.00)	−4.909	0.000
**Stigma**	54.50(10.00)	42.00 (19.00)	7.376	0.000	53.00 (12.00)	37.00 (18.00)	8.842	0.000
Social rejection	21.00 (4.00)	16.00 (8.00)	6.726	0.000	20.00 (5.00)	14.00 (7.00)	6.973	0.000
Financial insecurity	6.00 (2.00)	5.00 (3.00)	5.253	0.000	6.00 (2.00)	4.00 (2.75)	6.120	0.000
Internalized shame	12.00 (3.00)	9.00 (5.00)	5.596	0.000	12.00 (3.00)	8.00 (4.00)	7.027	0.000
Social isolation	16.00 (3.00)	12.00 (6.00)	8.330	0.000	15.00 (4.00)	11.00 (7.00)	8.145	0.000
**Self-efficacy**	22.14 ± 4.71	25.50 ± 5.19	−4.894	0.000	22.73 ± 4.99	27.13 ± 4.53	−6.567	0.000
**Optimism**	14.00 (4.00)	17.00 (3.00)	−6.938	0.000	15.00 (4.00)	18.00 (2.00)	−6.199	0.000
**Perceived stress**	20.00 (4.00)	15.00 (5.50)	8.696	0.000	19.00 (5.00)	14.00 (4.75)	9.244	0.000

Normal variables with homogeneous variances were expressed as M ± SD and analyzed by t test; Variables with non-normal or uneven variance were expressed by median (IQR) and analyzed by non-parametric test

*M* mean, *SD* standard deviation, *IRQ* Inter Quartile Range

### Predictors of anxiety symptoms and depressive symptoms

Stepwise Logistic regression analysis was conducted to identify the predictors of anxiety symptoms and depressive symptoms. Variables that were significantly associated with anxiety symptoms were included in the logistic regression analysis, including demographic variables (age and gender), clinical variables (distant metastasis), hope, stigma, self-efficacy, perceived social support, optimism and perceived stress. Multicollinearity diagnostic tests showed that there was multicollinearity between predictor variables. As shown in Table 3, the value of Tolerance< 0.2 or VIF > 5 indicated that there might be multicollinearity between variables- “social rejection” and “social isolation”- and other variables.

**Table 3:** Result 1 of Multicollinearity diagnostic tests on variables related to anxiety symptoms

Collinearity Statistics	Tolerance	VIF
(Constant)		
Age	0.865	1.156
Gender	0.911	1.098
Distant metastasis	0.836	1.196
Temporality and future	0.404	2.473
Positive readiness and expectancy	0.353	2.829
Interconnectedness	0.296	3.381
Family support	0.396	2.523
Friend support	0.374	2.673
Other support	0.286	3.491
Perceived stress	0.473	2.116
Optimism	0.523	1.911
Social rejection	0.185	5.398
Internalized shame	0.294	3.406
Financial insecurity	0.362	2.766
Social isolation	0.172	5.825

Tolerance < 0.2 or VIF > 5 indicated the possibility of multicollinearity among variables

As shown in Table 4, the values of Eigenvalue< 0.01 or Condition Index> 30 indicated that there might be 5–8 multicollinearity relations in those variables. Variance Proportions> 0.5 indicated that there might be multicollinearity between these variables, they were: “Optimism” and “Internalized shame”, “optimism” and “friend support”, “social rejection” and “social isolation”, “Temporality and future” and “other support”, “Positive readiness and expectancy” and “Interconnectedness”, “constant” and “perceived stress”.

**Table 4:** Result 2 of Multicollinearity diagnostic tests on variables related to anxiety symptoms

Dimension	Eigenvalue	Condition Index	Variance Proportions															
			(Constant)	Age	Gender	Distant metastasis	Temporality and future	Positive readiness and expectancy	Interconnectedness	Family support	Friend support	Other support	Perceived stress	Optimism	Social rejection	Internalized shame	Financial insecurity	Social isolation
1	15.164	1.000	0.00	0.00	0.00	0.00	0.00	0.00	0.00	0.00	0.00	0.00	0.00	0.00	0.00	0.00	0.00	0.00
2	0.360	6.494	0.00	0.00	0.01	0.00	0.00	0.00	0.00	0.00	0.01	0.00	0.01	0.00	0.00	0.01	0.02	0.01
3	0.118	11.345	0.00	0.69	0.05	0.00	0.00	0.00	0.00	0.00	0.01	0.00	0.00	0.00	0.00	0.00	0.00	0.00
4	0.109	11.780	0.00	0.03	0.62	0.00	0.00	0.00	0.00	0.00	0.01	0.00	0.03	0.00	0.00	0.00	0.01	0.00
5	0.057	16.351	0.00	0.00	0.24	0.06	0.00	0.00	0.00	0.00	0.00	0.00	0.37	0.00	0.01	0.01	0.06	0.00
6	0.053	16.849	0.00	0.00	0.03	0.45	0.00	0.00	0.00	0.01	0.01	0.01	0.05	0.00	0.00	0.04	0.05	0.00
7	0.036	20.549	0.00	0.11	0.00	0.02	0.00	0.01	0.01	0.00	0.15	0.01	0.02	0.06	0.00	0.12	0.27	0.00
8	0.026	24.213	0.00	0.03	0.00	0.23	0.01	0.01	0.01	0.00	0.18	0.01	0.08	0.04	0.01	0.06	0.36	0.03
9	0.016	30.408	0.00	0.01	0.01	0.10	0.06	0.01	0.03	0.24	0.10	0.09	0.00	0.03	0.01	0.06	0.04	0.11
10	0.016	31.132	0.00	0.00	0.00	0.00	0.04	0.01	0.01	0.00	0.01	0.00	0.01	0.29	0.09	0.50	0.07	0.12
11	0.013	34.066	0.00	0.00	0.00	0.00	0.02	0.01	0.01	0.12	0.26	0.03	0.02	0.47	0.11	0.17	0.08	0.01
12	0.010	38.903	0.01	0.00	0.01	0.00	0.00	0.03	0.01	0.24	0.00	0.13	0.02	0.00	0.36	0.00	0.00	0.44
13	0.008	43.824	0.00	0.00	0.00	0.00	0.17	0.06	0.02	0.15	0.23	0.48	0.00	0.00	0.21	0.00	0.00	0.18
14	0.007	46.652	0.00	0.00	0.01	0.00	0.51	0.12	0.16	0.14	0.01	0.19	0.01	0.03	0.09	0.00	0.01	0.03
15	0.005	57.022	0.05	0.02	0.01	0.03	0.03	0.69	0.44	0.10	0.00	0.04	0.01	0.00	0.03	0.01	0.03	0.05
16	0.002	80.175	0.94	0.11	0.01	0.10	0.16	0.06	0.31	0.00	0.01	0.00	0.36	0.07	0.07	0.01	0.00	0.01

Eigenvalue < 0.01, Condition Index > 30, Variance Proportions > 0.5 indicated the possibility of multicollinearity among variables

Then, stepwise Logistic regression was conducted (variables were entered in the regression analysis at *P* < 0.05 and removed from the model at *P* > 0.10) and results were shown in Table 5, social isolation dimension of stigma, optimism, and perceived stress were found to be the predictors of anxiety symptoms among patients with oral cancer.

**Table 5:** Stepwise Logistic regression analysis on results of anxiety symptoms(*n* = 230)

	β	S.E	Wals	*P*	OR(95%CI)
Social isolation	0.436	0.125	12.207	0.000	1.547(1.211,1.975)
Optimism	−0.276	0.100	7.676	0.006	0.759(0.624,0.922)
Perceived stress	0.217	0.066	10.844	0.001	1.243(1.092,1.414)
Constant	−5.814	3.780	2.366	0.124	0.03

Percentile 95% CIs for ORs are defined using the values that mark the upper and lower 2.5% of OR value

*SE* standard error, *CI* confidence interval

Variables that were significantly associated with depressive symptoms were included in the logistic regression analysis, including demographic variables (age, gender, marriage and smoking), clinical variables (distant metastasis), hope, stigma, self-efficacy, perceived social support, optimism and perceived stress. Multicollinearity diagnostic tests showed that there was multicollinearity between predictor variables. As shown in Table 6, the value of Tolerance< 0.2 or VIF > 5 indicated that there might be multicollinearity between variables- “social rejection” and “social isolation”- and other variables.

**Table 6:** Result 1 of Multicollinearity diagnostic tests on variables related to depressive symptoms

Collinearity Statistics	Tolerance	VIF
(Constant)		
Age	0.863	1.159
Gender	0.530	1.885
Distant metastasis	0.829	1.206
Smoking	0.490	2.042
Marriage	0.911	1.098
Temporality and future	0.398	2.515
Positive readiness and expectancy	0.351	2.850
Interconnectedness	0.294	3.407
Family support	0.395	2.531
Friend support	0.371	2.693
Other support	0.285	3.514
Perceived stress	0.458	2.184
Optimism	0.522	1.917
Social rejection	0.185	5.405
Internalized shame	0.289	3.455
Financial insecurity	0.354	2.823
Social isolation	0.171	5.837

Tolerance < 0.2 or VIF > 5 indicated the possibility of multicollinearity among variables

As shown in Table 7, the value of Eigenvalue< 0.01 or Condition Index> 30 indicated that there might be 5–8 multicollinearity relations. Variance Proportions> 0.5 indicated that there might be multicollinearity between these variables, they were: “smoking” and “gender”, “Optimism” and “Internalized shame”, “Optimism” and “Friend support”, “social rejection” and “social isolation”, “social isolation” and “other support”, “Temporality and future” and “other support”, “Positive readiness and expectancy” and “Interconnectedness”, “constant” and “perceived stress”.

**Table 7:** Result 2 of Multicollinearity diagnostic tests on variables related to depressive symptoms

Dimension	Eigenvalue	Condition Index	Variance Proportions																	
			(Constant)	Age	Gender	Distant metastasis	Smoking	Marriage	Temporality and future	Positive readiness and expectancy	Interconnectedness	Family support	Friend support	Other support	Perceived stress	Optimism	Social rejection	Internalized shame	Financial insecurity	Social isolation
1	17.027	1.000	0.00	0.00	0.00	0.00	0.00	0.00	0.00	0.00	0.00	0.00	0.00	0.00	0.00	0.00	0.00	0.00	0.00	0.00
2	0.366	6.820	0.00	0.00	0.01	0.00	0.00	0.00	0.00	0.00	0.00	0.00	0.01	0.00	0.01	0.00	0.00	0.01	0.02	0.01
3	0.169	10.044	0.00	0.01	0.15	0.00	0.12	0.00	0.00	0.00	0.00	0.00	0.00	0.00	0.01	0.00	0.00	0.00	0.00	0.00
4	0.117	12.068	0.00	0.69	0.00	0.00	0.00	0.00	0.00	0.00	0.00	0.00	0.02	0.00	0.00	0.00	0.00	0.00	0.00	0.00
5	0.068	15.779	0.00	0.03	0.01	0.05	0.06	0.03	0.00	0.00	0.00	0.00	0.01	0.01	0.19	0.00	0.01	0.01	0.05	0.01
6	0.054	17.755	0.00	0.01	0.12	0.27	0.01	0.00	0.00	0.00	0.00	0.01	0.02	0.01	0.16	0.00	0.00	0.02	0.06	0.00
7	0.044	19.677	0.00	0.08	0.20	0.12	0.16	0.00	0.00	0.00	0.00	0.00	0.07	0.00	0.04	0.02	0.00	0.07	0.10	0.00
8	0.034	22.401	0.00	0.02	0.01	0.26	0.00	0.30	0.00	0.00	0.00	0.00	0.03	0.00	0.00	0.00	0.00	0.05	0.25	0.01
9	0.027	25.313	0.00	0.04	0.31	0.11	0.44	0.12	0.00	0.01	0.00	0.00	0.11	0.00	0.02	0.05	0.00	0.07	0.09	0.00
10	0.019	29.858	0.00	0.00	0.02	0.01	0.01	0.37	0.01	0.01	0.01	0.03	0.18	0.00	0.19	0.07	0.04	0.00	0.27	0.04
11	0.016	32.790	0.00	0.01	0.01	0.06	0.04	0.01	0.13	0.03	0.04	0.17	0.05	0.11	0.00	0.07	0.01	0.01	0.00	0.03
12	0.016	33.085	0.00	0.00	0.00	0.01	0.01	0.02	0.00	0.00	0.00	0.03	0.00	0.01	0.03	0.26	0.07	0.54	0.05	0.20
13	0.013	36.174	0.00	0.00	0.00	0.00	0.00	0.01	0.02	0.02	0.01	0.13	0.28	0.03	0.01	0.43	0.10	0.19	0.06	0.02
14	0.010	42.095	0.01	0.00	0.03	0.00	0.02	0.03	0.01	0.02	0.01	0.27	0.00	0.13	0.06	0.00	0.38	0.00	0.00	0.41
15	0.008	46.576	0.00	0.00	0.00	0.00	0.01	0.00	0.13	0.06	0.02	0.13	0.22	0.49	0.00	0.00	0.26	0.00	0.00	0.22
16	0.007	49.562	0.00	0.00	0.00	0.00	0.00	0.00	0.49	0.14	0.17	0.16	0.01	0.18	0.02	0.03	0.06	0.00	0.01	0.02
17	0.005	61.277	0.02	0.03	0.03	0.02	0.02	0.00	0.02	0.67	0.56	0.08	0.00	0.04	0.00	0.00	0.02	0.01	0.03	0.04
18	0.002	93.641	0.97	0.09	0.10	0.09	0.10	0.10	0.18	0.04	0.18	0.00	0.00	0.00	0.26	0.05	0.05	0.01	0.00	0.00

Eigenvalue < 0.01, Condition Index > 30, Variance Proportions > 0.5 indicated the possibility of multicollinearity among variables

Then, stepwise Logistic regression was conducted (variables were entered in the regression analysis at *P* < 0.05 and removed from the model at *P* > 0.10) and results were shown in Table 8, marriage, positive readiness and expectancy dimension of hope, social isolation dimension of stigma, and perceived stress were found to be predictors of depressive symptoms among patients with oral cancer.

**Table 8:** Logistic regression analysis on results of depressive symptoms (*n* = 230)

	β	S.E,	Wals	*P*	OR(95%CI)
Marriage (Single/divorced/widow **VS** Married/ cohabitation	1.648	0.659	6.249	0.012	5.198(1.427,18.924)
Positive readiness and expectancy	−0.505	0.216	5.437	0.020	0.604(0.395,0.923)
Social isolation	0.314	0.133	5.558	0.018	1.368(1.054,1.776)
Perceived stress	0.273	0.075	13.146	0.000	1.314(1.134,1.524)
Constant	−5.747	4.949	1.349	0.245	0.003

Percentile 95% CIs for ORs are defined using the values that mark the upper and lower 2.5% of OR value

*SE* standard error, *CI* confidence interval

## Discussion

The current study explored the prevalence and predictors of anxiety symptoms and depressive symptoms in patients with oral cancer. The prevalence of anxiety symptoms in the current study was 36.96%, which was higher than previous researches [9]. The prevalence of depressive symptoms in the study was 65.21%, which was similar with the results in previous studies among cancer patients [9], and higher than a meta-analysis on the prevalence of depression in Chinese adults with cancer patients (54.9%) [8]. A recent research among patients with oral cancer [44] also confirmed the similar findings at different time points (at diagnosis, 1 month, and 3 months after treatment). This phenomenon is particularly obvious in patients with oral cancer due to facial deformity and dysfunction, and can be explained as the assumption that anxiety is likely to be caused by the on-the-spot sense of uncertainty, while depression by losing hope for the future and meaning of life.

As to the socio-demographic variables, it was surprising to find that married/cohabitation patients had a much higher risk of suffering from depressive symptoms than the unmarried group, which was different from previous studies [45, 46]. However, some population-related studies in China are similar to the results of this study [47, 48]. We speculate that this result maybe was partly due to the specificity of Chinese culture and the age of the patients. In China, “extended family” exists in a large number, that is, a family composed of three or even four generations, with a strong family concept, consanguinity and family ethics. Parents and children are always one family. Even when their children grow up, it is natural for them to pay for their children and serve them [49]. Married individuals usually have a more complete family life. In the current cultural background of China, family members usually get more care from their spouses and family members after they get sick. But at the same time, major diseases will bring more pressure to the whole family. The age of the patients in this study is in the year of “the old and the young”, which is the economic pillar of the family. The pressure of the family economy and the change of family order brought by the patients will inevitably bring more distress to the patients. Moreover, cancer is such a taboo topic in China that is easily associated with uninformed and misinformed social recognitions [50].

According to the results of logistic regression analysis, perceived stress was associated with both anxiety and depressive symptoms. Other researches [6, 51]suggested that the perceived stress impacted the depressive and anxiety symptoms of cancer patients through their mental adjustment. It could be explained by the fact that a cancer diagnosis is a stressful event for most individuals, and patients experience mental stress such as worries about prognosis and treatments, disruption of daily functions and survival time [52]. Hence, reducing stress may be considered a specific strategy to alleviate negative mood of patients with oral cancer for cancer specialized nurses and clinicians.

Stigma, especially the dimension of social isolation, was associated with both anxiety and depressive symptoms, which is consistent with previous studies [53–55]. Consequences of disease-related stigma were considered serious because it can not only arise psychological distress to patients, but also lead to poor health outcomes [56]. In this study, social isolation dimension was positively and significantly associated with depressive symptoms. Social isolation signifies a feeling of anomie in the traditional sociological sense, incorporating feelings of loneliness, inequality with others, and uselessness [33]. Patients with oral cancer are at an elevated risk of stigma because the cancer and its treatment often result in significant changes to physical appearance and functions. These changes occur in a highly visible and socially significant part of body and are associated with psychosocial impairment. As such, there is a vital need to address their perceived stigma when care to patients with oral cancer is delivered.

Hope is one of the positive coping resources for people experiencing difficult situations [18]. It has been found in this study that hope was a relative important protective factor for depressive symptoms among oral cancer patients; especially the positive readiness and expectancy dimension, which was set to measure affective-behavioral dimension of hope [57]. This finding suggested that patients with high level of hope were likely to manifest fewer depressive symptoms, which is consistent with other studies. A retrospective cohort study [58] showed that patients’ subjective hope for improvement can predict depression remission. Meisam Rahimipour [59] found that a high level of hope can protect those individuals’ renal failure from occurrence and the relapse of depression. Thus, possibly, enhancing the level of hope, especially “positive readiness and expectancy”, was one of the important ways to decrease the depressive symptoms of oral cancer patients in China.

Another positive coping resource, optimism, was found to be a relative important protective factor for anxiety symptoms among oral cancer patients. Optimism moderated the relationship between social support and anxiety, and there was a strong negative association between social support and anxiety for participants with low optimism [60]. Sanda Dolcos [61] provided biological structural evidence that increased gray matter volume (GMV) in left brain region protects against symptoms of anxiety through increased optimism. Higher levels of optimism were significantly associated with fewer anxiety and depressive symptoms, less hopelessness and better QOL [60]. Although optimism was a stable personality trait of a person, we can still do something to convert pessimism to optimism through some activities. Aussie optimism program (AOP) was a proven program that could improve the level of optimism effectively [62, 63].

Notably, optimism, but not hope, was associated with anxiety symptoms; hope, but not optimism, was associated with depressive symptoms. This result was similar with a study targeting patients with advanced cancer, including gastrointestinal cancer, colorectal cancer, lung cancer, or melanoma [22]. Although hope has been confirmed related to almost all health outcomes [64], it can be considered as the expectations for the future life after diagnosis. Additionally, optimism is more about cognition of the current life. Hence, results suggested that the greater hope, the less depressive symptoms; the more optimistic, the less anxiety symptoms. Thus, hope- or optimism-focused interventions can be taken into account to help alleviate specific aspects of psychological distress among patients with oral cancer in the future.

However, the current study results were not consistent with our hypothesis in that perceived social support and self-efficacy showed neither significant relations with anxiety symptoms nor with depressive symptoms. Therefore, further research is still needed to explore the exact mechanism of the two variables.

### Significance

The current study aims at identifying the possible influencing factors associated with anxiety and depressive symptoms in patients with oral cancer. The hypothetical socio-demographic and psychological variables were analyzed, resulting in significant results. This suggests that clinicians and nurses should make a complete assessment of patients’ information, especially their psychological status, at the time of pre-, peri, and post-discharge. In addition, it is now generally accepted that patients’ social, spiritual and psychological well-being are important parts of the multidisciplinary approach to the treatment of oral cancers. Results of our study suggest that intervention strategies to reduce perceived stress, stigma, especially social isolation, rebuild and enhance the level of optimism and hope, especially strategies to promote positive action, could be considered for health care organizations. Health education, psychotherapy, cognitive behavioral therapy, and supportive and group interventions have been reported effective in many studies. In this sense, our study further suggests the possibility that hope and optimism intervention may be especially worthy of use in oral cancer patients.

### Limitations

Due to the cross-sectional design, the causal relationship couldn’t be confirmed. Future research by means of longitudinal studies should be done to should assess whether positive resources or other positive behaviors have unintended effects on anxiety and depression by means of longitudinal studies. Besides, we only focused on the associations of anxiety/depressive symptoms with hope, stigma, self-efficacy, optimism, perceived stress and perceived social support; other factors which may be important to consider for depressive symptoms were not included. Moreover, the size of the sample is relatively small and a larger and multicenter sample is needed to improve the representativeness. Despite some limitations, our study provided some theoretical and clinical implications and suggested potentially better ways to reduce depressive symptoms through modifying both the negative and positive factors.

## Conclusions

After adjusting for demographic factors, perceived stress and social isolation of stigma were positively and significantly associated with both anxiety and depressive symptoms. Optimism was negatively and significantly associated with anxiety symptoms, and positive readiness and expectancy dimension of hope was negatively and significantly associated with depressive symptoms. However, perceived social support and self-efficacy had no significant relations with depressive symptoms. The communal predictors of anxiety and depressive symptoms in patients with oral cancer were levels of perceived stress and social isolation of stigma. In addition, optimism was a predictor of anxiety symptoms and hope was a predictor of depressive symptoms.

## Data Availability

The datasets supporting the conclusion of this article are included within the article. The underlying datasets are available from the corresponding author on reasonable request.

## Abbreviations

SAS: Zung Self-Rating Anxiety Scale
CES-D: The Center for Epidemiologic Studies Depression Scale
SIS: Social Impact Scale
HHI: Herth Hope Index
MSPSS: Multi- dimensional Scale of Perceived Social Support
LOT-R: Revised Life Orientation Test
PSS-10: Perceived Stress Scale-10
GSE: General Self-efficacy Scale
ANOVA: Analysis of Variance
BMI: Body Mass Index
SD: Standard Deviation
CI: Confidence Interval
